# ADDRESSING STRESS AMONG CAREGIVERS OF PEOPLE LIVING WITH DEMENTIA (PLWD) WITH BEHAVIORAL SYMPTOMS

**DOI:** 10.1093/geroni/igae098.0342

**Published:** 2024-12-31

**Authors:** Ana-Maria Vranceanu, Natalia Giraldo Santiago, Arden O’Donnel, Christine Ritchie

**Affiliations:** Massachusetts General Hospital, Boston, Massachusetts, United States; CHOIR-CASI, Boston, Massachusetts, United States; MGH Institute of Health Professions, Boston, Massachusetts, United States; Mass General Hospital and Harvard Medical School, Boston, Massachusetts, United States

## Abstract

Current stress management interventions for caregivers of PLWD are primarily focused on providing support or education targeting general caregiver stress. Here we showcase the development and refinement of MASC, a theoretically informed program that integrates emotional regulation skills (mindfulness, compassion and self-compassion) with practical strategies to manage stress from challenging behavioral symptoms in PLWD. First, we report on a mixed methods study (N=24 caregivers who endorse stress from PLWD’s behavioral symptoms; NIH stage 1A). Qualitative analyses provided a nuanced understanding of caregiver stress, highlighted the need for MASC, and showcased opportunities for tailoring of skills, program format and content. Quantitative analyses supported the need for the program and our theoretically informed conceptual model. Next, we report on an open pilot with exit interviews (N=10 caregivers; 2 groups; NIH stage 1A). MASC was feasible and acceptable (89% of eligible caregivers agreed to participate; 92% attended > 4/6 sessions; 70% reported at least moderate program satisfaction). Across the entire sample participation in MASC was associated with improvement in outcomes (stress, depression, and quality of life; ES=.3-.6) and mechanisms (self-compassion, compassion, and mindfulness; ES=.2-.7). Change in mechanisms was associated with change in outcomes, consistent with our conceptual model. Exit interviews supported the quantitative findings and provided recommendations for refinement and improvement. An NIH stage 1B pilot RCT of the refined MASC and protocol is ongoing. Integration of the Science of Behavior Change with the NIH stage model throughout this work will be detailed.

